# Complex body size trends in the evolution of sloths (Xenarthra: Pilosa)

**DOI:** 10.1186/s12862-014-0184-1

**Published:** 2014-09-10

**Authors:** Sara Raj Pant, Anjali Goswami, John A Finarelli

**Affiliations:** Department of Genetics, Evolution & Environment, University College London, Gower Street, London, WC1E 6BT UK; Department of Earth Sciences, University College London, Gower Street, London, WC1E 6BT UK; School of Biology & Environment Science, University College Dublin, Science Centre – West, Belfield, Dublin 4, Ireland; UCD Earth Institute, University of College Dublin, Belfield, Dublin 4, Ireland

**Keywords:** Ancestral character state reconstruction, Evolutionary rates, Fossils, Mammalia

## Abstract

**Background:**

Extant sloths present an evolutionary conundrum in that the two living genera are superficially similar (small-bodied, folivorous, arboreal) but diverged from one another approximately 30 million years ago and are phylogenetically separated by a radiation of medium to massive, mainly ground-dwelling, taxa. Indeed, the species in the two living genera are among the smallest, and perhaps most unusual, of the 50+ known sloth species, and must have independently and convergently evolved small size and arboreality. In order to accurately reconstruct sloth evolution, it is critical to incorporate their extinct diversity in analyses. Here, we used a dataset of 57 species of living and fossil sloths to examine changes in body mass mean and variance through their evolution, employing a general time-variable model that allows for analysis of evolutionary trends in continuous characters within clades lacking fully-resolved phylogenies, such as sloths.

**Results:**

Our analyses supported eight models, all of which partition sloths into multiple subgroups, suggesting distinct modes of body size evolution among the major sloth lineages. Model-averaged parameter values supported trended walks in most clades, with estimated rates of body mass change ranging as high as 126 kg/million years for the giant ground sloth clades Megatheriidae and Nothrotheriidae. Inclusion of living sloth species in the analyses weakened reconstructed rates for their respective groups, with estimated rates for Megalonychidae (large to giant ground sloths and the extant two-toed sloth) were four times higher when the extant genus *Choloepus* was excluded.

**Conclusions:**

Analyses based on extant taxa alone have the potential to oversimplify or misidentify macroevolutionary patterns. This study demonstrates the impact that integration of data from the fossil record can have on reconstructions of character evolution and establishes that body size evolution in sloths was complex, but dominated by trended walks towards the enormous sizes exhibited in some recently extinct forms.

**Electronic supplementary material:**

The online version of this article (doi:10.1186/s12862-014-0184-1) contains supplementary material, which is available to authorized users.

## Background

**Figure 1:**
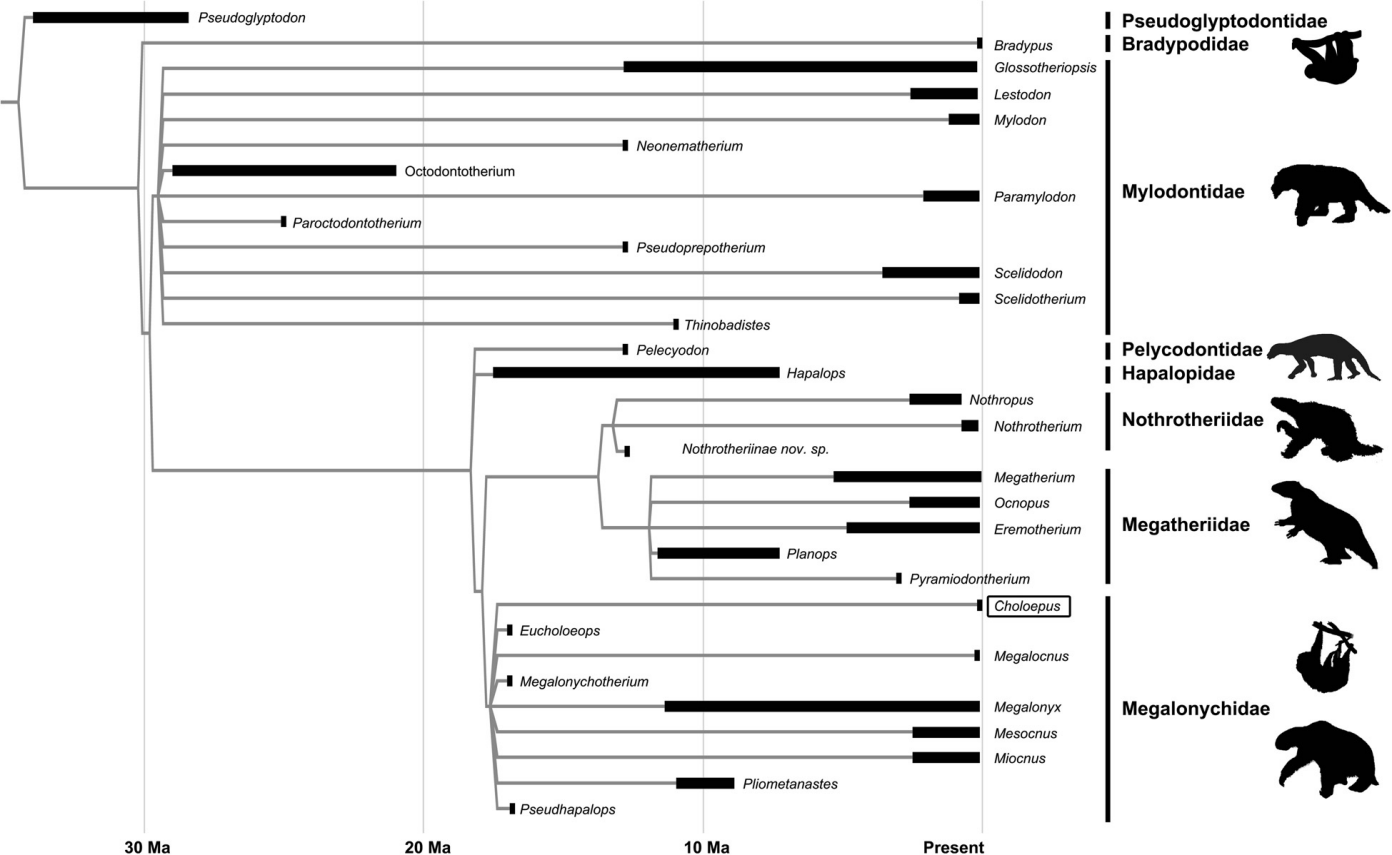
**Cladogram of phylogenetic relationships among sloth genera based on recent phylogenetic analyses** **[** 1,5,10 **].** Branch lengths are scaled to appearance events in the fossil record for each genus, with dark bars indicating the temporal ranges in the data set (Additional file 1: Table S1). Note that most genera contain multiple species and the analyses presented here were performed at the species-level. Families are indicated on the right. The topology of this cladogram was used in the compilation of taxonomic groups for the body size analysis presented here. Silhouettes are modified from phylopic.org.

Living sloths, folivorans (Xenarthra, Pilosa), comprise six South American species in the genera *Bradypus* and *Choloepus* (each placed in its own family: Bradypodidae and Megalonychidae, respectively). In contrast to the present-day low diversity of sloths, the fossil record reveals that this group was far more diverse in the past, with more than 50 known species distributed among eight families see [1–3]. The Pleistocene megafaunal extinction events (~2.6 million to 11,700 years ago) reduced sloth generic diversity by approximately 90% [4]. Recent phylogenetic analyses reconstruct bradypodids and megalonychids as rather distantly related (Figure 1), implying that their superficially similar suspensory posture and locomotion have been derived convergently [1,5], possibly as a result of constraints imposed by fossorial adaptations in early Xenarthrans [6].

**Figure 2:**
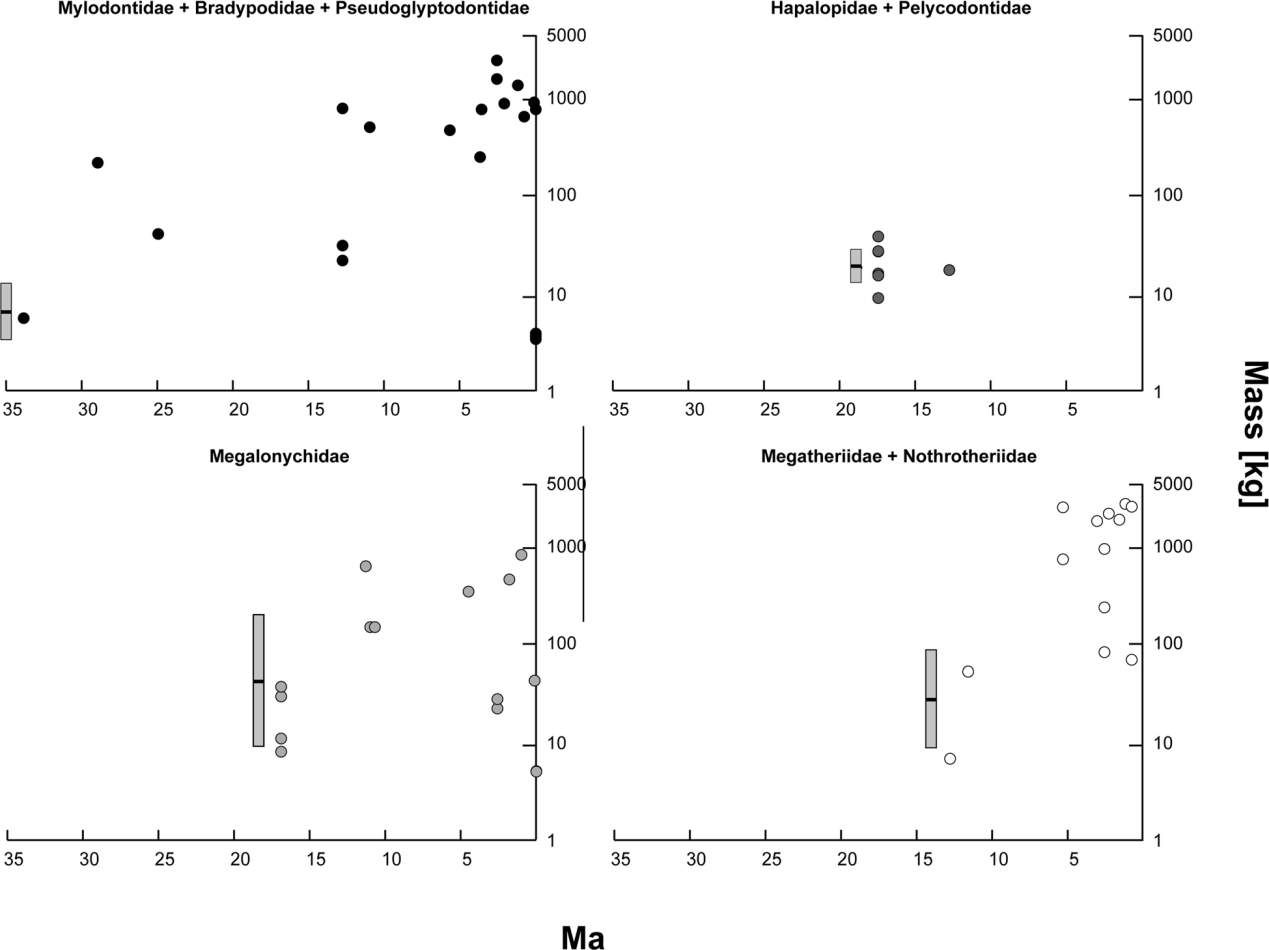
**Body sizes for sloth species plotted at the FAE for each species in Additional file 1: Table S1.** Species are arranged in plots as a function of the taxonomic grouping used in the analyses. The reconstructed body size distributions, based on the all-taxa, weighted-average model, are presented in the box plot just before the FAE of the oldest species in each group (black bar: ancestral mean [μ_0_] black bar, grey box: standard deviation [σ_0_^½^]).

Accurate reconstructions of character evolution are crucial for proper inference of underlying macroevolutionary processes. Yet, extant taxa may not always be adequate proxies for modelling past evolutionary processes [7–9]. In the case of sloths, the six living species are all folivorous, fully arboreal, with a very narrow range of body sizes; extant species mean body masses are between 3.5 and 5.5 kg (see Additional file 1: Table S1). In contrast, extinct sloths exhibited a range of ecological diversity, with ground-dwelling, semi-arboreal, aquatic, and bipedal forms, as well as inferred diets that include grazers and even omnivores [10]. Additionally, extinct sloths exhibited a wide range of body sizes unobserved in the Recent, with species in the late Paleogene (~35 million years ago) genus *Pseudoglyptodon* estimated to have been approximately six kg [3], whereas the Late Pleistocene species *Megatherium americanum* reached as much as 3800 kg [11]. Indeed, very large body sizes (estimated masses in excess of 1000 kg) are observed in at least four different sloth families (Figure 2; Additional file 1: Table S1).

Body mass is of broad significance in evolutionary biology, macroecology, and macroevolution, as it is fundamentally linked to many ecological and life-trait variables [12–15]. Thus, a better understanding of the evolution of this character will enhance our understanding of the evolution of life history and ecology among extant, as well as extinct, taxa [16–18]. This is particularly important when considering currently depauperate clades that were once species-rich, such as sloths [3], as the fossil record holds unique information that can markedly change and improve reconstructions of character evolution [7–9,19–21].

Here, we reconstruct sloth body size evolution using body mass data for both living and fossil species, demonstrating the impact of fossil data on our understanding of body size evolution for this group. We model the evolution of sloth body size through time using a “family”-level phylogeny and a general time-variable model [8,22,23] for analyzing continuous character evolution in clades lacking fully-resolved phylogenies. Our model structure estimates ancestral average body mass and the variance of the ancestral body mass distribution, and allows either parameter to increase or decrease as a function of time. In addition, the model can be applied to an entire group, or to constituent subgroups based on phylogenies [8,22]. We calculated the log-likelihood fits of 340 models to the body size data for 57 species of extinct and extant sloths (Additional file 1: Table S1), and evaluated support among competing models using the finite sample Akaike Information Criterion (AIC_c_) [24,25] (see Methods for details).

## Results and discussion

Of the 340 models examined, eight fell within 2 LnL units of the optimal score, forming a well-constrained “credible set” of models (Additional file 1: Table S4). Six of these models estimated separate model parameters for each of the four major sloth subgroups, while Models #315 and #320 estimated a single trended random walk (fully parameterized) model for a combined clade of Megalonychidae, Megatheriidae, and Nothrotheriidae (Additional file 1: Tables S4 and S5). The predominant reconstructed evolutionary modes in the credible set were trended random walks (43% of subgroup reconstructions: Additional file 1: Table S5), followed by driven trends and Brownian motion (23% each). Stasis was reconstructed in only three cases (10%). Reconstructed modes were not evenly distributed across sloth subgroups. All but one of the best-supported models supported a trended walk for the basal group (Mylodontidae + Bradypodidae + Pseudoglyptodontidae), with the other model supporting a driven trend. The Hapalopidae + Pelycodontidae grouping returned Brownian motion in all but two model structures, with the remaining models supporting trended walks. Megatheriidae + Nothrotheriidae results were evenly split, with four models supporting a driven trend towards large body sizes, while the other four supported a trended random walk (two in combination with Megalonychidae). Finally, Megalonychidae, which includes the modern two-toed sloths, showed varied results, with the only observed reconstructions of evolutionary stasis occurring in this clade.

We calculated model-averaged parameter estimates [26] for the credible set of 8 models (Table 1). The model-averaged estimates for the mean drift parameter (α) and the variance inflation term (β) vary greatly among the four groups, reflecting different inferred modes of body size evolution. The basal group displayed high reconstructed values for the mean drift parameter (α), emphasizing a pronounced trend to larger body sizes. The inferred mean body size increase over the observed range of this group is approximately 11.6 kg/million year (hereafter, My). The model-averaged estimate of β was also high, indicating a trend to increased variance.

**Table 1:** **Model-averaged parameter estimates for sloth body size**

		**Model-averaged parameter estimates**			
		**All taxa included**			**Extant taxa excluded**
**Taxonomic subgroup**	**μ0**	**L. 2LnL CI**	**Est.**	**U. 2LnL CI**	
Megalonychidae		3.086	3.924	4.767	3.214
Megatheriidae + Nothrotheriidae		2.599	3.368	4.111	3.144
Hapalopidae + Pelycodontidae		2.961	2.961	2.961	2.986
Mylodontidae + Bradypodidae + Pseudoglyptodontidae		1.822	1.938	2.055	2.796
	**σ0**	**L. 2LnL CI**	**Est.**	**U. 2LnL CI**	
Megalonychidae		1.272	2.526	6.09	0.557
Megatheriidae + Nothrotheriidae		0.749	1.609	4.433	1.211
Hapalopidae + Pelycodontidae		0.171	0.171	0.171	0.166
Mylodontidae + Bradypodidae + Pseudoglyptodontidae		0.301	0.528	1.021	4.943
	**α**	**L. 2LnL CI**	**Est.**	**U. 2LnL CI**	
Megalonychidae		0.012	0.037	0.061	0.163
Megatheriidae + Nothrotheriidae		0.233	0.317	0.402	0.372
Hapalopidae + Pelycodontidae		−0.006	−0.006	−0.006	−0.011
Mylodontidae + Bradypodidae + Pseudoglyptodontidae		0.076	0.12	0.163	0.121
	**β**	**L. 2LnL CI**	**Est.**	**U. 2LnL CI**	
Megalonychidae		0.029	0.069	0.173	0.212
Megatheriidae + Nothrotheriidae		0.037	0.084	0.212	0.110
Hapalopidae + Pelycodontidae		−0.036	−0.036	−0.036	−0.035
Mylodontidae + Bradypodidae + Pseudoglyptodontidae		0.141	0.244	0.476	−0.146

Parameter estimates based on all taxa in the Additional file 1: Table S1 with associated model-averaged 2LnL CI’s are given for each parameter. In addition, model-averaged estimates for parameters from the analysis excluding extant taxa (Additional file 1: Table S6) are given.

Both of the linear drift parameters were slightly negative, with CI’s excluding zero, for the Hapalopidae + Pelycodontidae group in the model-averaged estimates (Table 1), indicating decrease in mean body size and reduced variance through time for this group, although the absolute value of these terms was quite low. Thus, although stasis was not supported for this group, the model-averaged parameters do not indicate large changes in the body size distribution through time for Hapalopidae + Pelycodontidae (inferred change in mean body mass was only −0.11 kg/My for this group).

The model-averaged estimate for α for Megatheriidae + Nothrotheriidae was strongly positive (Table 1), indicating a pronounced trend to increased size in this group, with an inferred increase in mean body mass of 129 kg/My (range: 42 to 384 kg/My, based on estimated CIs for α; Table 1) for this clade. Estimates for β were generally low, although positive. Both α and β were low, but also positive, for the Megalonychidae, with an inferred increase in body size of about 2.6 kg/My (range: 0.7 to 5.3 kg/My) across its range.

Despite the coarse phylogenetic resolution currently available for fossil sloths, the combination of fossil and extant taxa into a single analysis has permitted reconstruction of an interesting and surprisingly complex pattern of body size evolution. A single set of rate parameters cannot describe the evolution of body size in this clade, and reconstructing these patterns would not have been possible absent data from the fossil record. Our analysis support trended walks as the predominant model of sloth body size evolution, with driven trends and random walks receiving some support as well. All four sloth subgroups were separately parameterized in 6 of the 8 best-supported models, reflecting distinct body size distributions for each group. Moreover, each clade is reconstructed with markedly difference ancestral body masses (Additional file 1: Table S6) that increase from earlier to later diverging groups, demonstrating a general trend towards larger body size through their evolution.

Previous studies of mammal body size evolution focusing only on extant taxa have reconstructed small-bodied ancestral body sizes for sloths, with a low variances [27], which is not surprising given the observed body size distribution in extant sloths. The problem is that, in the particular case of sloths, the extant sample represents such a minor fraction of their evolutionary diversity, both taxonomically and morphologically, that inferences drawn from living taxa are unlikely to accurately reflect true evolutionary processes. Indeed, inferences based on a depauperate clade of small-bodied folivores would be hard-pressed to reconstruct anything along the lines of giant megatheriid ground sloths [9,19,28,29]. This issue is clearly reflected in the ancestral body mass estimates for all of the sub-groups when fossil taxa are incorporated in the analysis (Additional file 1: Table S4).

To estimate the effect of the anomalous extant sloths on reconstructions of evolutionary trends in this clade, we reanalysed the dataset excluding the two genera of extant sloths. Here, only two models were strongly supported (Additional file 1: Table S6), and the model-averaged results were largely similar for the two groups with no extant membership (Megatheriidae + Nothrotheriidae and Hapalopidae + Pelycodontidae, see Table 1). However, model-averaged estimates of α and β for Megalonychidae indicated a substantial trended random walk to larger body size with larger variance for this clade. Estimates for both parameters were approximately four times greater than those estimated when including extant *Choloepus* and fall outside the estimated CIs for both paramters (Table 1). Thus, not only would an analysis of the extant taxa alone provide an oversimplification of sloth body size evolution, but extant sloths appear to actively obscure an otherwise strong signal in the fossil record.

The ability of fossil data to improve inferences of character evolution is a function of the amount of the data the fossil record can bring to bear on the reconstructions. While sloths were more diverse in the past than they are today, sloths have never been a highly speciose clade. The available sample size, even incorporating fossil data is somewhat small (57 taxa spanning ~35 million years, Additional file 1: Table S2). More specifically, it is important to note that the method we employ here is unable to resolve finer scale trends within individual lineages, and more complex processes of body size evolution may be masked by a lack of phylogenetic resolution, low sample size, and the resulting reduction in resolving power, particularly for clades such as Megalonychidae. For example, the sole extant megalonychid genus, *Choloepus*, is the smallest megalonychid taxon (mean mass of ~5.5 kg), nested within a clade with a mean body mass of 236 kg and a reconstructed ancestral body mass of 50 kg. It is therefore almost certain that an isolated shift to smaller body size occurred in within megalonychids, but we currently lack the fossil data to resolve this trend.

Another caveat of this method is that it is reliant on *a priori* groupings. For example, the extant genus *Bradypus* is one of the earliest-diverging lineages in the phylogeny (Figure 1) [1,5] and is not itself nested within a large-bodied clade. It is possible that the bradypodids diverged from other sloths prior to the onset of the trended walk towards larger body size that is reconstructed for the basal group in which it was included, and is therefore not part of this larger pattern. If we were to group Bradypodidae and Pseudoglyptodontidae in a basal group apart from the Mylodontidae, this would result in a partition of six species in two genera: one genus (two species) from >30 Mya and one Recent genus (four species). This very large gap between essentially two data points will almost inevitably resolve as a trended walk, simply as an artefact of the temporal distance between the genera, rather than a real evolutionary trend. In an attempt to bridge the 30 million year gap between these families, we included Mylodontidae, a speciose family of medium- to large-bodied sloths spanning nearly the entire temporal gap between pseudoglyptodontids and bradypodids. However, it is possible that the reconstruction of a trended body size increase in the basal group is actually a punctuated event across the small-to-large gap between pseudoglyptodontids and bradypodids on the one hand and mylodontids on the other.

If we analyse Mylodontidae on its own, we reconstruct a strong positive trend in mean body size, comparable to that reconstructed for the entire basal group: the model-averaged estimate for α was 0.113 for Mylodontidae, as compared to 0.120 for the basal group in Table 1. However, reconstructed β for Mylodontidae alone was −0.162, suggesting a variance decrease through time in this large-bodied clade when analysed separately. Similarly, ancestral mass reconstructions for the basal group combine the small-bodied Bradypodidae and Pseudoglyptodontidae (median body mass ~4 kg) with the much larger Mylodontidae (median mass of ~920 kg). As such, the reconstructed ancestral mass of approximately 66 kg (Table 1) for the basal group seems rather large, in light of the branching order of the cladogram (Figure 1). Analysing Mylodontidae to the exclusion of the small bodied basal clades produces estimates of the ancestral mean body mass more in accord with observed masses in each group (~6 kg for the basal group and ~31 kg for mylodontids). As such, there was likely an increase in body size along the branch between bradypodids + pseudoglyptodontids and mylodontids. Whether this was a punctuated event or a trend is ambiguous given the current data, but we can be confident of a trend of increasing body size within the Mylodontidae, and that this is not an artefact of our *a priori* grouping.

## Conclusion

Despite these caveats, which stem from long temporal gaps in the fossil record of some sloth groups (e.g., Bradypodidae) and the lack of a fully resolved phylogenetic tree for living and extinct sloths, our analysis greatly expands the reconstructions of the evolutionary processes that led to gigantism in multiple sloths clades. All but two of the top models estimated parameters separately for the maximum number of sloth subgroups, and those that did not combined only two of the sub-groups. This points to the ability of even modest amounts of fossil data to uncover hidden complexity in character evolution for groups such as sloths, see also [7]. Rather than the long-term stasis reconstructed from extant representatives, sloth body size evolution was governed predominantly by directional trends towards enormous body sizes that are difficult to infer from modern taxa.

A great deal of debate has centred on the importance of fossil data to accurate analysis of macroevolutionary patterns, and recent studies have demonstrated that excluding extinct taxa can result in misleading reconstructions of diversity dynamics [30–33] and trait evolution [7–9,20,34]. This issue can be particularly problematic for clades that were previously diverse, but are presently species-poor, such as tuataras, hyaenas, or sloths. One recently-described method [7] for including data from fossil representatives as node priors into analyses that predominantly sample extant taxa presents a promising avenue for circumventing issues of misleading patterns among extant taxa, as well as issues of poor phylogenetic resolution and patchiness in data coverage for many fossil groups. Applying such an approach to a clade with as few living relatives as extant sloths will likely prove more challenging than for groups with more balanced distributions of living and extinct species, but future work should expand upon these approaches to take advantage of the strengths of the extant record (well-resolved phylogenies, genetic and developmental information, potentially complete trait, ecology, and diversity data), as well as the fossil record (primary observation of evolutionary tempo and additional character data for many taxa unobserved in the present day).

## Methods

Quantifying evolutionary rates by measuring change in average phenotype through time can potentially oversimplify inferences of evolutionary trends [35–39], as apparent trends can arise from changes in the mean and/or the variance of the distribution of trait values [35,40–42]. Accurate modelling of evolutionary trends must account for potential change in both average phenotype and its variance through time [22,23,27,43]. We modelled sloth body size evolution using published mass estimates for extinct and extant taxa using a potentially changing normal distribution for log-transformed sloth body mass data (Additional file 1: Table S1).

We employed a general time-variable model [8], expanding on Hunt’s [22,23] approach for analyzing continuous character evolution in time series to account for changes in both average phenotype and variance, while allowing for analysis of trends in clades lacking fully-resolved phylogenies, such as sloths. We modelled sloth body sizes as events in a time series of species first appearance events (FAEs). The log-likelihood of a normal distribution of mean (M) and variance (V) is given by:

1 $$ LogL\left(M,V\Big|x\right)\propto -\frac{1}{2}\ast \log \left[V\right]-\frac{{\left(x-M\right)}^2}{\left(2\ast V\right)} $$

[8,22] we allow M and V to be potentially variable through time, such that M(t) and V(t):

2a $$ M(t)=\alpha \ast t+{\mu}_0, $$

2b $$ V(t)=\beta \ast t+{\sigma_0}^2, $$

where *t* is the elapsed time along a branch [8,22].

In equations 2a and 2b, μ_0_ and σ_0_ are estimates of the ancestral mean and variance, respectively, describing an initial body mass distribution for a group. The linear trend terms, α and β, describe the drift in mean body mass and the variance inflation factor of a Brownian motion model, respectively [8]. Together, α and β describe the how the body size distribution changes from its root condition (μ_0_, σ_0_^2^) through time. This allows for the construction of a variety of models including evolutionary stasis [44] (α = β = 0), Brownian Motion [45,46] (α = 0, β ≠ 0), driven/active trends [35,42] (α ≠ 0, β = 0), and trended random walks [22,23] (α ≠ 0, β ≠ 0). Parameterizations of model forms are given in Additional file 1: Table S2 see also [8].

We applied these four model types to a dataset of body sizes and FAEs for 57 species of fossil and extant sloth collected from the literature (Additional file 1: Table S1). Model parameters were optimized using the Solver tool in Excel 2010 (Microsoft Corp., Redmond, WA) to maximise the objective (likelihood value) using the Simplex LP solving method. Parameters were first estimated for all sloth taxa as a single group, defined by a global set of parameters for all species. We then explored further models (Additional file 1: Table S3), partitioning sloth species into subgroups following a backbone phylogeny (Figure 1), which was constructed from the topologies of several recent phylogenetic analyses [1,5,10]. In total, we evaluated 340 distinct model structures (see Additional file 1: Table S3).

For each model structure, we computed a log-likelihood score (“Total Model LnL” in Additional file 1: Table S3), corresponding to the sum of the subgroup log-likelihoods (“Submodel LnL”). We used AIC_c_ to select among models for increased fit, while correcting for model complexity see [26]. Total model parameterization is the sum of the number of parameters fit to each submodel parameters for each sloth subgroup. We computed the AIC_c_-adjusted model log-likelihood to select the models that best fit the data after being penalized for the number of estimated parameters [26,47].

3 $$ LogL(Model)\propto -\frac{1}{2}\ast \left( AI{C}_c(Model)- MinAI{C}_c\right) $$

[26], we used a cutoff of 2 LnL units [48,49] to construct a credibility interval of around the best fit model to demarcate significant difference in support among models. For the models within 2LnL units of the optimal score, we estimated confidence intervals (CIs) around the parameter estimates, by calculating the upper and lower values for each parameter necessary to reduce the submodel likelihood by 2LnL.

A weighted-average model was calculated for the set of models within 2 LnL units of the optimal score, by calculating the proportional likelihoods of each model in this set, and then multiplying the proportional likelihood for each model by each parameter estimate and the upper and lower CI estimates. These were then summed across all of the models to create weighted-average estimates for each model parameter [26].

## Additional file

Additional file 1: Table S1. Data for the 57 sloth species used in this study. Family membership, body mass, first and last appearance events (FAE, LAE), and sources for body mass estimates are provided. **Table S2.** Evolutionary model structures and the estimated parameters for each. Evolutionary stasis occurs when a plesiomorphic mean and variance for the body size distribution do not change though time. Brownian motion indicates linear change through time in the variance of the distribution with no change in the mean, while driven trends describe distributions with constant variance and linear change in the mean. Trended walks are models that reconstruct linear change in both mean and variance through time. “***” indicates that the given parameter is not estimated for that particular model type (see: Finarelli and Goswami 2013 [8]). **Table S3.** Models descriptions, parameterizations and likelihoods. AICc scores were used to calculate the parameter-corrected likelihoods for each model. **Table S4.** Summary of model likelihoods and parameter estimates for the optimal model and the 8 models within 2LnL of the optimal score. Models are in order of descending likelihood, with the model numbers corresponding to those given in Table S3. *** indicates a parameter that was excluded from the particular submodel structure. Means are in Ln(kg) and variances are Ln(kg^2), while α and β are in Ln(kg)/My and Ln(kg^2)/My, respectively. Upper and lower CI's corresond to the 2LnL limit for submodel parameter estimates. See Methods. **Table S5.** Graphical summary of the inferred modes of evolution implied by the set of models within 2 log-likelihood units of the opimal model. As with Table S4, models are ordered in descending log-likelihood score. **Table S6.** Parameter estimates for sloth body size for the analysis excluding extant sloth species (Choleopus and Bradypus). Only two models were found within 2 LnL units of the optimal score. Model numbers correspond to the same structure from Table S3.

## References

[1] Gaudin TJ (2004). Phylogenetic relationships among sloths (Mammalia, Xenarthra, Tardigrada): the craniodental evidence. Zool J Linn Soc.

[2] McKenna MC, Bell SK (1997). Classification of Mammals Above the Species Level.

[3] Croft DA (2000). Archaeohyracidae (Mammalia: Notoungulata) from the Tinguiririca Fauna, central Chile, and the evolution and paleoecology of South American mammalian herbivores. PhD thesis.

[4] Steadman DW, Martin PS, MacPhee RDE, Jull AJT, McDonald HG, Woods CA, Iturralde-Vinent M, Hodgins GWL (2005). Asynchronous extinction of late Quaternary sloths on continents and islands. Proc Natl Acad Sci.

[5] Pujos F, De Iuliis G, Argot C, Werdelin L (2007). A peculiar climbing Megalonychidae from the Pleistocene of Peru and its implication for sloth history. Zool J Linn Soc.

[6] Nyakatura JA (2012). The convergent evolution of suspensory posture and locomotion in tree sloths. J Mamm Evol.

[7] Slater GJ, Harmon LJ, Alfaro ME (2012). Integrating fossils with molecular phylogenies improves inference of trait evolution. Evolution.

[8] Finarelli JA, Goswami A (2013). Potential pitfalls of reconstructing deep time evolutionary history with only extant data, a case study using the Canidae (Mammalia, Carnivora). Evolution.

[9] Finarelli JA, Flynn JJ (2006). Ancestral state reconstruction of body size in the Caniformia (Carnivora, Mammalia): the effects of incorporating data from the fossil record. Syst Biol.

[10] Pujos F, Gaudin TJ, De Iuliis G, Cartelle C (2012). Recent advances on variability, morpho-functional adaptations, dental terminology, and evolution of sloths. J Mamm Evol.

[11] Vizcaíno SF, Bargo MS, Cassini GH (2006). Dental occlusal surface area in relation to body mass, food habits and other biological features in fossil Xenarthrans. Ameghiniana.

[12] McNab BK, Montgomery GG (1985). Energetics, population biology, and distribution of Xenarthrans, living and extinct. The evolution and ecology of armadillos, sloths, and vermilinguas.

[13] Damuth J (1987). Interspecific allometry of population density in mammals and other animals: the independence of body mass and population energy- use. Biol J Linn Soc.

[14] Isaac NJB, Jones KE, Gittleman JL, Purvis A (2005). Correlates of species richness in mammals: body size, life history, and ecology. Am Nat.

[15] Bielby J, Mace GM, Bininda-Emonds ORP, Cardillo M, Gittleman JL, Jones KE, Orme CDL, Purvis A (2007). The fast-slow continuum in mammalian life history: an empirical reevaluation. Am Nat.

[16] Fariña RA (1996). Trophic relationships among Lujanian mammals. Evol Theor Rev.

[17] Bargo MS, Vizcaíno SF, Archuby FM, Blanco RE (2000). Limb bone proportions, strength and digging in some Lujanian (Late Pleistocene-Early Holocene) mylodontid ground sloths (Mammalia, Xenarthra). J Vert Paleo.

[18] McDonald HG (2005). Paleoecology of extinct Xenarthrans and the Great American biotic interchange. Bull Fla Mus Nat Hist.

[19] Oakley TH, Cunningham CW (2000). Independent contrasts succeed where ancestor reconstruction fails in a known bacteriophage phylogeny. Evolution.

[20] Slater GJ (2013). Phylogenetic evidence for a shift in the mode of mammalian body size evolution at the Cretaceous-Palaeogene boundary. Methods in Ecology and Evolution.

[21] Albert JS, Johnson DM, Knouft JH (2009). Fossils provide better estimates of ancestral body size than do extant taxa in fishes. Acta Zoolog.

[22] Hunt G (2006). Fitting and comparing models of phyletic evolution: random walks and beyond. Paleobiology.

[23] Hunt G (2007). The relative importance of directional change, random walks, and stasis in the evolution of fossil lineages. Proc Natl Acad Sci.

[24] Hurvich CM, Tsai C-L (1989). Regression and time series model selection in small samples. Biometrika.

[25] Akaike H, Petrov BN, Csaki F (1973). Information theory as an extension of the maximum likelihood principle. Second International Symposium on Information Theory.

[26] Burnham KP, Anderson DR (2002). Model Selection and Multimodel Inference: A Practical Information-Theoretic Approach.

[27] Venditti C, Meade A, Pagel M (2011). Multiple routes to mammalian diversity. Nature.

[28] Garland T, Harvey PH, Ives AR (1992). Procedures for the analysis of comparative data using phyogenetically independent contrasts. Syst Biol.

[29] Cunningham CW (1999). Some limitations of ancestral character-state reconstruction when testing evolutionary hypotheses. Syst Biol.

[30] Liow LH, Fortelius M, Bingham E, Lintulaakso K, Mannila H, Flynn L, Stenseth NC (2008). Higher origination and extinction rates in larger mammals. Proc Natl Acad Sci.

[31] Liow LH, Quental TB, Marshall CR (2010). When can decreasing diversification rates be detected with molecular phylogenies and the fossil record?. Syst Biol.

[32] Liow LH, Finarelli JA: **A dynamic global equilibrium in carnivoran diversification over 20 million years.** *Pro Biol Sci* 2014, **281**(1778):20132312. 10.1098/rspb.2013.2312 PMC3906931 24452020

[33] Finarelli JA, Badgley C (2010). Diversity dynamics of Miocene mammals in relation to the history of tectonism and climate. Pro Biol Sci.

[34] Finarelli JA, Flynn JJ (2009). Brain size evolution and sociality in Carnivora. Proc Natl Acad Sci.

[35] Wagner PJ (1996). Contrasting the underlying patterns of active trends in morphologic evolution. Evolution.

[36] Wang SC (2005). Accounting for unequal variances in evolutionary trend mechanisms. Paleobiology.

[37] Alroy J (1998). Cope's rule and the dynamics of body mass evolution in North American fossil mammals. Science.

[38] Jablonski D (1997). Body-size evolution in Cretaceous molluscs and the status of Cope's rule. Nature.

[39] Alroy J (2000). Understanding the dynamics of trends within evolving lineages. Paleobiology.

[40] Gould SJ (1988). Trends as changes in variance - a new slant on progress and directionality in evolution. J Paleontol.

[41] Stanley SM (1973). An explanation for Cope's rule. Evolution.

[42] McShea DW (1994). Mechanisms of large-scale evolutionary trends. Evolution.

[43] Solow AR, Wang SC (2008). Some problems with assessing Cope's Rule. Evolution.

[44] Roopnarine PD (2001). The description and classification of evolutionary mode: a computational approach. Paleobiology.

[45] Felsenstein J (1985). Phylogenies and the comparative method. Am Nat.

[46] McShea DW, Brandon RN (2010). Biology's First Law: The Tendency for Diversity and Complexity to Increase in Evolutionary Systems.

[47] Burnham KP, Anderson DR (2004). Multimodel inference: Understanding AIC and BIC in model selection. Soc Meth Res.

[48] Edwards AWF (1992). Likelihood: Expanded Edition.

[49] Royall RM (1997). Statistical evidence: a likelihood paradigm.

